# Cardiac microstructural alterations measured by echocardiography identify sex-specific risk for heart failure

**DOI:** 10.1136/heartjnl-2022-320876

**Published:** 2022-06-09

**Authors:** Alan Kwan, Emmanuella Demosthenes, Gerran Salto, David Ouyang, Trevor Nguyen, Chike C Nwabuo, Eric Luong, Amy Hoang, Ewa Osypiuk, Plamen Stantchev, Elizabeth H Kim, Pranoti Hiremath, Debiao Li, Ramachandran Vasan, Vanessa Xanthakis, Susan Cheng

**Affiliations:** 1 Department of Cardiology, Smidt Heart Institute and Biomedical Imaging Research Institute, Cedars-Sinai Medical Center, Los Angeles, California, USA; 2 Framingham Heart Study, Framingham, Massachusetts, USA; 3 Division of Cardiology, Department of Medicine, Johns Hopkins University, Baltimore, Maryland, USA; 4 Ronin Institute, Montclair, New Jersey, USA; 5 Departments of Medicine, Biostatistics, and Epidemiology, Boston University Schools of Medicine and Public Health, Boston, Massachusetts, USA

**Keywords:** Heart Failure, Echocardiography, Cardiomyopathies

## Abstract

**Objective:**

Established preclinical imaging assessments of heart failure (HF) risk are based on macrostructural cardiac remodelling. Given that microstructural alterations may also influence HF risk, particularly in women, we examined associations between microstructural alterations and incident HF.

**Methods:**

We studied N=2511 adult participants (mean age 65.7±8.8 years, 56% women) of the Framingham Offspring Study who were free of cardiovascular disease at baseline. We employed texture analysis of echocardiography to quantify microstructural alteration, based on the high spectrum signal intensity coefficient (HS-SIC). We examined its relations to incident HF in sex-pooled and sex-specific Cox models accounting for traditional HF risk factors and macrostructural alterations.

**Results:**

We observed 94 new HF events over 7.4±1.7 years. Individuals with higher HS-SIC had increased risk for incident HF (HR 1.67 per 1-SD in HS-SIC, 95% CI 1.31 to 2.13; p<0.0001). Adjusting for age and antihypertensive medication use, this association was significant in women (p=0.02) but not men (p=0.78). Adjusting for traditional risk factors (including body mass index, total/high-density lipoprotein cholesterol, blood pressure traits, diabetes and smoking) attenuated the association in women (HR 1.30, p=0.07), with mediation of HF risk by the HS-SIC seen for a majority of these risk factors. However, the HS-SIC association with HF in women remained significant after adjusting for relative wall thickness (representing macrostructure alteration) in addition to these risk factors (HR 1.47, p=0.02).

**Conclusions:**

Cardiac microstructural alterations are associated with elevated risk for HF, particularly in women. Microstructural alteration may identify sex-specific pathways by which individuals progress from risk factors to clinical HF.

**WHAT IS ALREADY KNOWN ON THIS TOPIC**

- Sexual dimorphism is seen in the development of heart failure. Changes in cardiac microstructure can be identified using echocardiographic texture analysis.

**WHAT THIS STUDY ADDS**

- Cardiac microstructural alterations are associated with incident heart failure in women, and the effects of many traditional risk factors are mediated by these microstructural alterations.

**HOW THIS STUDY MIGHT AFFECT RESEARCH, PRACTICE OR POLICY**

- The study findings suggests that detectable changes in cardiac microstructure are related to heart failure risk, especially in women, and further investigating these microstructural changes could improve our understanding of how women and men tend to develop and present with heart failure differently.

## Introduction

**Figure 1:**
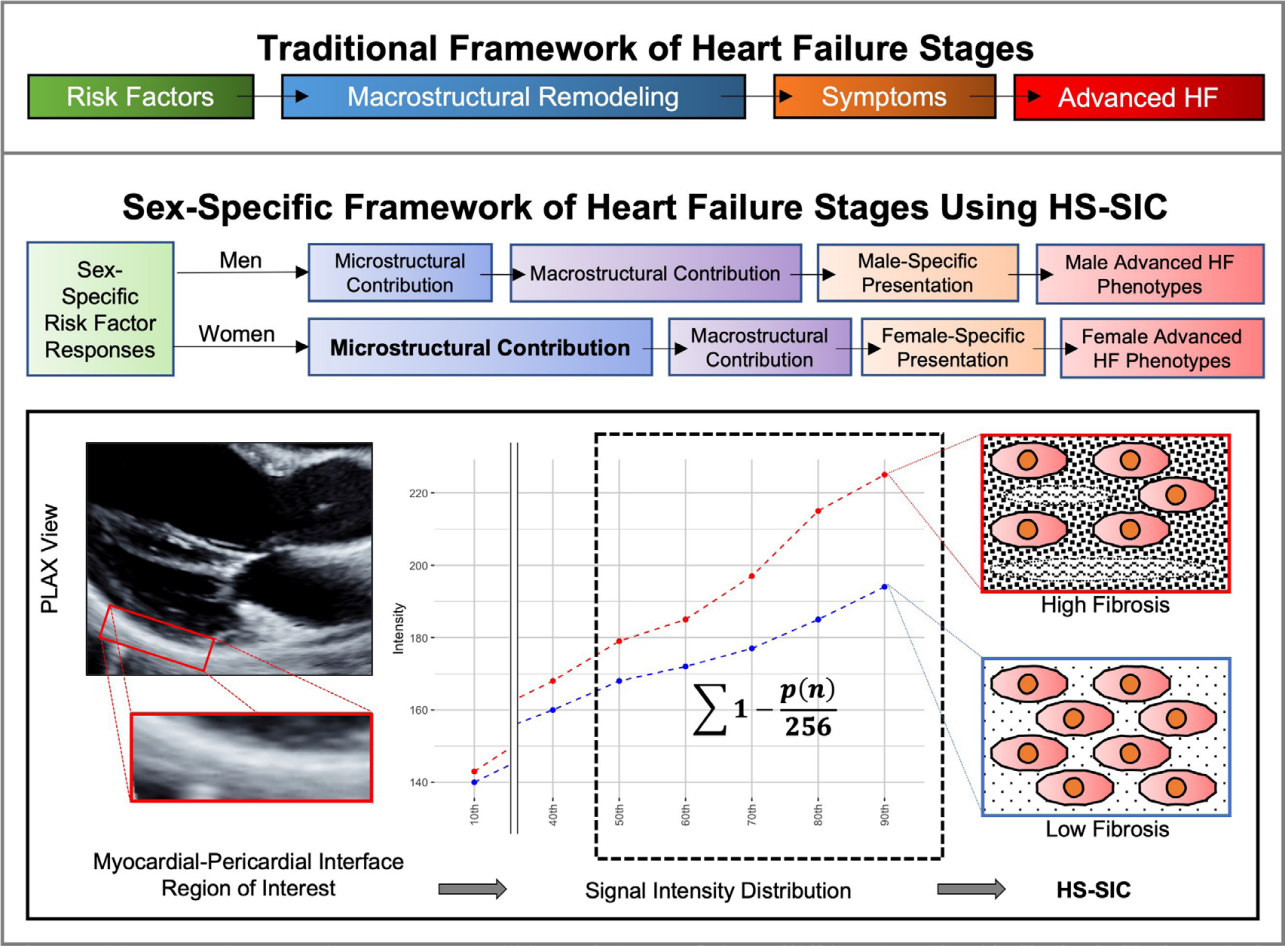
Traditional frameworks of heart failure (HF) stages may insufficiently capture early microstructural alterations that can reveal sex-specific features of HF risk. Therefore, as part of an a priori sex-based analytical framework, we used the high-spectrum signal intensity coefficient (HS-SIC) to identify microstructural alterations and examine their potential sex-specific associations with incident HF. The HS-SIC, a measure previously related to myocardial fibrosis, is calculated using a B-mode parasternal long axis (PLAX) view, with a region-of-interest (ROI) placed at the myocardial–pericardial interface at the level of the mitral leaflet tips. The ROI is analytically processed to provide a distribution of signal intensities, with the HS-SIC calculated as the sum of 1 − the normalised intensity. Higher HS-SIC levels are associated with increased myocardial fibrosis.

The well-described stages of heart failure (HF) continue to be essential for guiding clinical assessment and management across the spectrum of disease risk.^1^ While originally defined and validated in a sex-agnostic manner, there are consistently observed pathophysiological differences between women and men at every HF stage and growing recognition that risk assessments could benefit from sex-specific considerations.^2 3^ Observed sex differences in HF risk appear mediated by sexual dimorphism in response to stressors occurring at the cellular/microstructural and microvascular level.^4 5^ Conventional imaging approaches rely on macrostructural cardiac abnormalities visible by standard measures of wall thickness, mass and function, thus focusing on measures of remodelling that may underestimate risk in women.^6 7^ Accordingly, methods for identifying microstructural cardiac alterations could provide insights regarding divergence of HF clinical manifestations between sexes. To this end, we investigated whether an ultrasonic measure of cardiac microstructure could offer preclinical information regarding risk for overt HF incidence in a large community-based cohort (figure 1). We conducted both sex-pooled and a priori sex-specific analyses to examine whether characterisation of myocardial alterations beyond macrostructure could elucidate HF risk while accounting for sex as a biological variable. We designed this study with the aim to improve precision of HF risk assessment through the evaluation of a novel imaging biomarker.

## Methods

### Study sample

Our study sample included participants of the Framingham Offspring cohort who received routine examinations approximately every 4 years. The Framingham Offspring cohort is a second-generation cohort study started in 1971, which includes the children of the original Framingham Heart Study and their spouses.^8^ Of all study participants who attended the eighth examination between 2005 and 2008 (N=3021), we included for the current study those who completed standardised echocardiography with adequate quality images for analysis^9^ and did not have pre-existing cardiovascular disease (coronary heart disease, HF or stroke) at the time of echocardiography to assess for the incidence of new-onset cardiovascular events (N=2511) (online supplemental figure 1). The study complies with the Declaration of Helsinki.

10.1136/heartjnl-2022-320876.supp1 Supplementary data

### Echocardiography and signal intensity coefficient analysis

We applied an ultrasonic method for characterising cardiac tissue microstructure that is referred to as the ‘high spectrum signal intensity coefficient’ (HS-SIC). Our study staff performed transthoracic echocardiography and analysis using standardised techniques.^10–14^ We selected transthoracic B-mode parasternal long axis views of the left ventricle at end-diastole. We analysed static images (8-bit DICOM or JPG) using the ImageJ software platform (V.1.46, National Institutes of Health, Bethesda, Maryland, USA) to identify a prespecified region of interest of the pericardium adjacent to the mid-to-basal inferolateral myocardial wall. For the region of interest, standardised analysis generated a hierarchical distribution of signal intensities (0–255).^11–14^ We calculated high spectrum SIC as ∑1-(p(n)/256), where p(n) are the 50th, 60th, 70th, 80th and 90th percentiles of signal intensity within the region of interest. The SIC measure has been associated with myocardial interstitial fibrosis and microfiber disarray in murine models of afterload stress from aortic banding, blood pressure elevation in patients with hypertension, myocardial interstitial fibrosis in hypertrophic cardiomyopathy genetic carriers, and risk factor burden and lipid derangements in metabolic syndrome.^11–14^ Traditional ultrasound-based approaches to assessing cardiac microstructure, including backscatter analysis methods, have relied on calculating the average values of measurable grayscale values with variable results shown across prior studies.^15 16^ Extending from conventional backscatter methods, the HS-SIC measure assesses both values and distribution of the signal intensities. The region of interest for measuring HS-SIC is focused on the myocardial–pericardial interface given that the echocardiographic signal reflected by the pericardium is dependent on the transmission through the proximal and adjacent myocardium in the parasternal long axis view (figure 1). As shown in prior work,^11–14^ this method offers the ability to discriminate between myocardial phenotypes while serving as a relatively accessible and widely applicable post-acquisition method given that the parasternal long axis view is routinely acquired in a standardised manner as part of most echocardiographic studies. Previous measurements of inter-reader and intra-reader reproducibility have been reported at 0.89 and 0.90, respectively, and the measurements have also demonstrated high reproducibility across gain settings.^11^

We considered the continuous HS-SIC variable as our primary exposure variable of interest. Along with the HS-SIC as the primary measure of microstructural alteration, we also conducted parallel analyses of relative wall thickness (RWT) given its established role as a measure of early macrostructural alteration (ie, concentric remodelling) in the absence of overt cardiac disease.^17 18^

### Outcome of interest

We specified the primary outcome as incident HF using the well-established Framingham definition: presence of two major or one major plus two minor criteria. Major criteria include paroxysmal nocturnal dyspnoea or orthopnoea, distended neck veins, rales, radiographic cardiomegaly, pulmonary oedema, S3, hepatojugular reflux and weight loss on diuretic therapy; minor criteria include bilateral ankle oedema, night cough, dyspnoea on exertion, hepatomegaly, pleural effusion, radiographic pulmonary vascular redistribution, decrease in vital capacity and tachycardia. As previously described, a panel of three experienced physicians adjudicated all new HF diagnoses via standardised medical records review.^19^

### Statistical analysis

All continuous variables were normalised by their respective SD. In cross-sectional analyses, we used multivariable linear regression to evaluate the association of clinical risk factors (independent variables: age, sex, body mass index (BMI), total cholesterol/high-density lipoprotein (HDL), systolic blood pressure, antihypertensive treatment, diabetes and smoking) with HS-SIC (dependent variable) in the overall sample as well as by sex.

In prospective analyses, we used multivariable-adjusted Cox proportional hazards models to examine the relation of HS-SIC with time to new-onset HF in the total sample with and without incorporating sex interaction terms. We tested the assumption of proportionality of hazards for all independent variables using scaled Schoenfeld residuals. Age alone failed the assumption and, thus, was stratified into four groups defined by quartiles (ie, fourths) for all analyses. We also investigated the shape of the relationship between HS-SIC and incident HF and found the test for non-linearity in restricted cubic spline Cox models to be non-significant. For the main exposure variable of interest, we considered HS-SIC as a continuous variable given that modelling HS-SIC as a categorical variable led to slightly inferior overall model fit parameters. In addition to formal tests of interaction, we conducted a priori prespecified sex-specific analyses. For the primary analyses, the base model considered HS-SIC as a continuous variable alone; model 1 additionally included sex and age; model 2 additionally adjusted for antihypertensive medication use, given the known correlation of SIC with hypertension^11^; and model 3 additionally adjusted for standard cardiovascular risk factors (BMI, systolic blood pressure, diabetes, smoking and total cholesterol to HDL ratio) as traits also known as having the potential to drive the development or progression of cardiac tissue alterations. In secondary analyses, we included RWT as an additional potential confounder (given its role as an established marker of early cardiac macrostructural alterations) in both the sex-pooled and sex-specific multivariable-adjusted Cox models.

Recognising there is interest in understanding how cardiac microstructural abnormalities could contribute to the progression from clinical risk factors to HF, we also conducted analyses of HS-SIC as a potential mediator of incident HF risk. Specifically, for the clinical traits previously established as risk factors for HF, we examined whether any of their associations with incident HF was mediated by the HS-SIC measure.^14^ As done in prior mediation analyses for incident HF,^20^ we used a standardised coefficient model to assess the extent to which HS-SIC mediated the association of each established risk factor (eg, diabetes) with incident HF.^21^

We conducted all statistical analyses using R (V.3.6.1) with Rstudio (1.2.5019) and defined statistical significance as a two-tailed pvalue<0.05.

### Patient and public involvement

Patients and the public were not involved in the development of this study.

## Results

The study sample included 2511 participants, aged 65.7±8.8 years and including 56% women (table 1). Over a follow-up period of 7.4±1.7 years, there were 94 incident HF events, including 57 (61%) in men and 37 (39%) in women. Conventional echocardiographic parameters were predominantly within normal range, with only 34 (1%) of participants with left ventricular ejection fraction (LVEF) less than 50%, and only 30 (1%) of participants with E/e’ greater than 14. In cross-sectional analyses at baseline, HS-SIC was directly associated with multiple risk factors in the pooled sex sample including advanced age, male sex, greater BMI, higher total cholesterol to HDL ratio, use of antihypertensive medication therapy and presence of diabetes. The HS-SIC was inversely associated with LVEF (p=0.01) and appeared directly associated with E/e’ (p=0.07). In analyses of women, results were similar to those in the total sample with age, BMI, total cholesterol to HDL ratio, antihypertensive therapy, diabetes and LVEF significantly associated with HS-SIC; in addition, a direct association with higher systolic blood pressure was present. In men, results were similar with the exceptions of the systolic blood pressure association, which was not significantly associated with HS-SIC, and E/e’ was directly associated with HS-SIC (table 2).

**Table 1:** Clinical characteristics of participants overall and by sex

	Total	Women	Men
N (%)	2511	1414 (56)	1097 (43)
Age (years)	65.7±8.8	65.8±8.7	65.5±8.8
Body mass index (kg/m^2^)	28.1±5.3	27.6±5.8	28.7±4.5
Systolic blood pressure (mm Hg)	128±17.1	128±17.4	129±16.6
Diastolic blood pressure (mm Hg)	73.9±9.94	72.6±9.68	75.6±9.99
Total cholesterol/HDL ratio	3.47±1.05	3.27±0.95	3.74±1.11
Diabetes (%)	289 (12)	121 (8.6)	168 (15)
Smoker (%)	217 (8.6)	135 (9.5)	82 (7.5)
eGFR (mL/min/1.73m^2^)	81.2±18.2	79.7±18.5	83.1±17.5
Antihypertensive treatment (%)	1122 (45)	583 (41)	539 (49)
Lipid-lowering treatment (%)	981 (39)	498 (35)	485 (44)
Glucose-lowering treatment (%)	189 (7.5)	89 (6.3)	100 (9.1)
Left ventricular ejection fraction (%)	68%±7%	69%±6%	66%±7%
E/e’ measurement	6.99±2.20	7.37±2.31	6.49±1.95

eGFR, estimated glomerular filtration rate; HDL, high-density lipoprotein.

**Table 2:** Age-adjusted and sex-adjusted associations of high spectrum signal intensity coefficient with clinical risk factors and echocardiographic markers

	Total sample			Women			Men		
	Est.	SE	P value	Est.	SE	P value	Est.	SE	P value
Female sex*	−0.43	0.04	<0.0001	–	–		–	–	
Age (per SD)*	0.16	0.02	<0.0001	0.14	0.02	<0.0001	0.18	0.03	<0.0001
Body mass index (per SD)	0.34	0.02	<0.0001	0.31	0.02	<0.0001	0.39	0.03	<0.0001
Total/HDL cholesterol (per SD)	0.15	0.02	<0.0001	0.18	0.07	<0.0001	0.13	0.03	<0.0001
Systolic blood pressure (per SD)	0.04	0.02	0.05	0.08	0.03	<0.01	−0.02	0.03	0.64
Antihypertensive treatment	0.21	0.04	<0.0001	0.25	0.05	<0.0001	0.16	0.06	0.01
Diabetes	0.40	0.06	<0.0001	0.47	0.09	<0.0001	0.35	0.09	<0.0001
Smoking	0.03	0.07	0.66	0.06	0.08	0.46	−0.02	0.12	0.85
LVEF (per SD)	−0.01	0.003	0.01	−0.01	0.004	0.02	−0.01	0.005	0.16
E/e’ measurement (per SD)	0.02	0.01	0.07	0.01	0.01	0.55	0.04	0.02	0.03

*Models including sex were adjusted for age only, and models including age were adjusted for sex only.

HDL, high-density lipoprotein; LVEF, left ventricular ejection fraction.

In prospective analyses of the sex-pooled sample, HS-SIC was associated with incident HF with an HR of 1.67 per 1-SD increment in HS-SIC (p<0.0001). Unadjusted cumulative incidence curves comparing individuals in the top fourth of HS-SIC values to those in the lower three-fourths, in total and sex-stratified samples, demonstrate the cumulative pattern of events and validity of the proportional hazards assumption (online supplemental figure 2). After adjustment for age and sex, the association between HS-SIC and incident HF remained statistically significant with an HR of 1.31 (p=0.04), but addition of other risk factors as covariates rendered the association statistically non-significant (table 3). The risk factor with the greatest magnitude of effect in attenuating the overall association of HS-SIC with incident HF was BMI, with similar effects seen for antihypertensive medication use; notably, attenuation of risk was more evident in men than in women.

**Table 3:** Associations of HS-SIC with incident heart failure, overall and by sex

	Total sample				Women			Men		
	HR*	95% CI	P value	Sex interaction P	HR*	95% CI	P value	HR*	95% CI	P value
Model 0	1.42	1.20 to 1.68	<0.0001	0.042	1.67	1.29 to 2.16	<0.0001	1.16	0.92 to 1.47	0.22
Model 1	1.20	1.01 to 1.44	0.04	0.047	1.46	1.13 to 1.90	<0.01	1.04	0.82 to 1.32	0.72
Model 2	1.18	0.98 to 1.41	0.07	0.07	1.37	1.05 to 1.79	0.02	1.03	0.81 to 1.32	0.78
Model 3	1.04	0.85 to 1.26	0.72	0.16	1.30	0.98 to 1.73	0.07	0.88	0.68 to 1.13	0.32

Model 0: HS-SIC alone.

Model 1: HS-SIC+age + sex.

Model 2: Model 1+antihypertensive medication use.

Model 3: Model 2+body mass index, systolic blood pressure, diabetes, smoking and total/high-density lipoprotein cholesterol ratio.

*Estimates are per 1-SD for HS-SIC.

HS-SIC, high spectrum signal intensity coefficient.

Following analyses suggesting interactions between sex and HS-SIC on incident HF risk (table 3), we conducted sex-specific analyses. In women, HS-SIC was significantly associated with incident HF in models 1 and 2 with a trend towards statistical significance in model 3 (table 3). Notably, the magnitudes of HF risk observed in women appeared greater than those seen in the total sample for all models. In men, HS-SIC was not associated with incident HF in any model. Sex-specific results were similar in analyses that adjusted for LVEF or E/e’ in addition to age, whereby the HS-SIC was associated with HF risk in women (HR 1.51 (95% CI 0.11 to 0.72), p=0.01 after adjusting for LVEF, and HR 1.34 (95%CI 0.00 to 0.58), p=0.05 after adjusting for E/e’) but not in men (p=0.67).

In secondary analyses, we examined the effect of including both RWT and HS-SIC as predictor variables included together in all models (table 4). We also assessed the effect of RWT alone in all models (table 5). With the addition of RWT, the magnitude of effects for HS-SIC became more prominent across all models, reaching statistical significance for model 2 in the total sample, and for model 3 in sex-specific analyses of women. The effect of RWT in analyses of RWT alone (table 5) and in combination with HS-SIC (table 4) had similar results. Notably, we observed a consistent sexual dimorphism in results of analysing RWT that was reverse of that seen in analyses of HS-SIC: whereas HS-SIC associations with incident HF were more prominent in women than in men, RWT associations with incident HF were more prominent in men than in women.

**Table 4:** Associations of high spectrum signal intensity coefficient and relative wall thickness with incident heart failure, overall and by sex

	High spectrum SIC			Relative wall thickness*		
	HR	95% CI	P value	HR	95% CI	P value
Total sample						
Model 0	1.44	1.19 to 1.74	<0.001	1.42	1.21 to 1.67	<0.0001
Model 1	1.25	1.03 to 1.52	0.02	1.21	1.02 to 1.45	0.03
Model 2	1.23	1.01 to 1.50	0.04	1.17	0.97 to 1.40	0.10
Model 3	1.10	0.89 to 1.35	0.38	1.13	0.95 to 1.36	0.17
Women						
Model 0	1.76	1.31 to 2.37	<0.001	1.06	0.73 to 1.53	0.76
Model 1	1.61	1.20 to 2.17	<0.01	0.83	0.56 to 1.23	0.35
Model 2	1.54	1.13 to 2.10	<0.01	0.78	0.53 to 1.17	0.23
Model 3	1.47	1.06 to 2.03	0.02	0.77	0.51 to 1.16	0.21
Men						
Model 0	1.19	0.92 to 1.53	0.18	1.53	1.27 to 1.85	<0.0001
Model 1	1.10	0.86 to 1.42	0.45	1.35	1.12 to 1.64	<0.01
Model 2	1.09	0.83 to 1.41	0.51	1.32	1.09 to 1.61	<0.01
Model 3	0.95	0.72 to 1.25	0.71	1.29	1.06 to 1.58	0.01

Model 0: High spectrum signal intensity coefficient and relative wall thickness.

Model 1: High spectrum signal intensity coefficient, relative wall thickness, age and sex.

Model 2: Model 1+antihypertensive medication use.

Model 3: Model 2+body mass index, systolic blood pressure, diabetes, smoking, and total/high-density lipoprotein cholesterol ratio.

*Estimates are per 1-SD for high spectrum SIC and per 1-SD for relative wall thickness (SD=0.0634).

SIC, signal intensity coefficient.

**Table 5:** Associations between relative wall thickness and incident heart failure, overall and by sex

	Total sample				Women			Men		
	HR	95% CI	P value	Sex interaction P	HR	95% CI	P value	HR	95% CI	P value
Model 0	1.48	1.27 to 1.73	<0.0001	0.151	1.17	0.83 to 1.65	0.39	1.55	1.28 to 1.86	<0.0001
Model 1	1.23	1.04 to 1.46	0.02	0.101	0.91	0.62 to 1.33	0.62	1.35	1.12 to 1.64	<0.01
Model 2	1.18	0.99 to 1.41	0.07	0.093	0.83	0.57 to 1.23	0.36	1.33	1.09 to 1.61	<0.01
Model 3	1.14	0.95 to 1.36	0.16	0.075	0.81	0.55 to 1.19	0.28	1.30	1.06 to 1.58	0.01

Estimates are per 1-SD for relative wall thickness (SD=0.0634).

Model 0: Relative wall thickness alone.

Model 1: Relative wall thickness and sex stratified by age.

Model 2: Model 1+antihypertensive medication use.

Model 3: Model 2+body mass index, systolic blood pressure, diabetes, smoking and total/high-density lipoprotein cholesterol ratio.

In analyses relating established clinical risk factors with incident HF, we observed that the effects of sex, BMI, systolic blood pressure, antihypertensive medication use and diabetes each appeared mediated by the HS-SIC (table 6). In effect, the magnitude of risk association between each of these risk factors and incident HF was significantly mediated by increased HS-SIC. For instance, our results indicate that a 10% proportion of the total effect of diabetes on HF risk may be explained by measured alteration in myocardial microstructure as represented by increase in HS-SIC (table 6).

**Table 6:** Mediation by HS-SIC on the association of risk factors with incident HF

	Mediation by high intensity signal intensity coefficient								
	Direct effect			Mediated effect** (ie, indirect effect)			Total effect		
	Estimate	SE	P value	Estimate	SE	P value	Estimate	SE	P value
Age	0.0359	0.0048	<0.001	0.0014	0.0007	0.053	0.0374	0.0048	<0.001
Sex	−0.0206	0.0086	0.010	−0.0054	0.0020	0.010	−0.0260	0.0082	0.001
Body mass index	0.0106	0.0043	0.014	0.0038	0.0016	0.019	0.0145	0.0044	0.001
Total/HDL cholesterol	0.0028	0.0045	0.54	0.0024	0.0009	0.007	0.0052	0.0043	0.24
Systolic blood pressure	0.0128	0.0042	0.002	0.0013	0.0005	0.012	0.0141	0.0041	0.001
Antihypertensive treatment	0.0407	0.0083	<0.001	0.0036	0.0015	0.017	0.0442	0.0083	<0.001
Diabetes	0.0538	0.0172	0.002	0.0060	0.0025	0.019	0.0598	0.0172	0.001
Smoking	−0.0244	0.0093	0.007	−0.0012	0.0010	0.26	−0.0256	0.0093	0.005

*The mediated effect can be considered the proportion of the total magnitude of association (ie, total effect) attributable to the mediator, in this case HS-SIC. For example, results shown in the table above suggest that HS-SIC is a significant mediator of the association between diabetes and incident HF, whereby 10% (indirect effect/total effect=0.006/0.060=0.10) of the total magnitude of risk observed for diabetes in relation to HF (total effect=0.060) is attributable to an increase in HS-SIC (indirect effect=0.006).

HDL, high density lipoprotein; HF, heart failure; HS-SIC, high spectrum signal intensity coefficient.

## Discussion

Cardiac microstructural alterations, like microvascular alterations, have been relatively understudied due to the limited number of accessible methods for non-invasively quantifying these findings in humans. Nonetheless, such discrete abnormalities appear important for understanding heterogeneity in cardiovascular disease manifestations pertaining to sex differences.^22^ Indeed, when we used the sensitive HS-SIC method for quantifying myocardial microstructure across a large community-based cohort, we observed not only that this measure of microstructural alteration corresponded with HF risk but that it was predominantly associated with risk in women and not in men. Conversely, in parallel analyses of RWT as an established parameter of early cardiac remodelling, we observed that this macrostructural measure of preclinical risk was associated with incident HF more prominently in men than in women. These findings together underscore the potential of cardiac microstructural measures, such as the HS-SIC, to offer both mechanistic and prognostic information regarding the development and incidence of HF—particularly in women—and in a manner that complements existing macrostructural measures.

Prior studies have reported on sex differences in risk factor trajectories,^3 5^ cardiac responses to stressors^23^ and clinically manifest HF phenotypes.^2^ While there are multiple pathways by which sex dimorphism could arise, cardiac microstructural differences may be related to increased myocardial fibroblast activity and subsequent collagen accumulation in women compared with men—a finding reported from murine studies as well as in the explanted hearts of older women compared with similarly aged men.^24^ In both women and men at risk for HF, microstructural changes will necessarily precede macrostructural abnormalities and are expected to mediate the effects of clinical factors—as we observed in the current study. In fact, we found that increased HS-SIC mediated up to 10% of the association seen between a risk factor such as diabetes and incident HF. In turn, the association between HS-SIC and HF risk was attenuated by the presence of cardiometabolic risk factors but much less so in women than in men, potentially related to a greater and more persistent female compared with male sensitivity to cardiometabolic stressors.^25^ It has long been recognised that in the setting of chronic hypertension, women tend to develop a concentric remodelling response while men tend to develop the morphologically more overt eccentric remodelling response.^26^ More recently, cardiac magnetic resonance studies have indicated that women compared with men consistently exhibit a greater degree of diffuse myocardial microstructural alteration, assessed using the extracellular volume (ECV) measure and representing interstitial fibrosis.^22 27^ The extent to which our ultrasonic measure of microstructural alteration also represents interstitial fibrotic change is not yet clear, although we have previously reported high correlation between elevated SIC and ECV measures.^13^ Nonetheless, our results along with known sex differences in HF phenotypes and trajectories^28^ may suggest that the paths from risk factors to structural abnormalities to clinical HF—typified as the transition from stage A to stage B and eventual stage C HF—are associated with myocardial tissue level microstructural alterations to a greater degree in women than in men, with relative preservation of ventricular morphology, at least during the early phases of preclinical disease progression. Our findings could also be due to sex differences in the temporality of risk exposures on the heart which may be more or less accelerated in women compared with men, causing our single timepoint analysis to represent different stages of disease progression between the sexes. Sex-specific temporal trends analyses of repeated measures should be considered for future studies.

There have been multiple alternative approaches regarding echocardiographic texture analysis within the published literature. Although the integrated backscatter techniques we attempt to improve on account for most of the historical literature, deep learning approaches have been recently reported.^29 30^ With the expansion in deep learning technology, we expect that most will outperform the HS-SIC, given its simplicity. Nonetheless, the simplicity may offer concrete benefits. Due to technical variation in routine echocardiographic image acquisition, our method may avoid overfitting to acquisition parameters, the focused region of interest may avoid noise from unremarkable regions, and no model training and intensive computing is required. Nonetheless, we present our method not as a definitive echocardiographic measure of myocardial fibrosis but as a simple approach that may reveal underlying insights regarding the pathophysiology of HF.

Several limitations of our study merit consideration. Initial histological validation for fibrosis was performed in non-human models wherein post-mortem cardiac analysis could be performed immediately after imaging. Thus, it remains possible that the HS-SIC is not a measure of myocardial fibrosis but is capturing signals associated with fibrosis that independently lead to HF. Our finding of sex-specific associations relating HS-SIC with incident HF should be considered hypothesis generating until they can be validated in separate and ideally larger-sized cohorts with ample events occurring within each sex. Accordingly, if validated, our results could be related to well-described sex differences in the relative risk for HF with preserved compared with reduced ejection fraction (HFpEF and HFrEF).^28^ Given that our sample size provided insufficient statistical power for analysing subtypes of incident HF, follow-up studies are also needed to further investigate sex-specific relations of microstructure with HFpEF versus HFrEF. Since we studied a community-based cohort of ambulatory adults who were relatively free of major comorbidities at the time of echocardiography, generalisability to clinical care setting remains unknown and warrants future investigation. Notwithstanding these limitations, strengths of the study included a community-based study sample under longitudinal surveillance for events, with exposures assessed using standardised protocols and clinical outcomes all systematically reviewed and adjudicated.

In summary, we applied an accessible ultrasonic method for quantifying alterations in cardiac microstructure in a large community-based cohort, and we found that variations in this microstructural measure distinguished persons at risk for developing clinical HF—particularly women. Additional studies are needed to validate our findings in other cohorts and evaluate whether assessments of cardiac microstructure might augment efforts to more precisely predict HF risk especially in women. Follow-up investigations are also required to determine whether early detectable alterations in myocardial microstructure may be considered as future targets for therapeutic intervention to alleviate the burdens of HF morbidity and mortality affecting both sexes.

## Data Availability

Data are available in a public, open access repository. Data are available upon reasonable request. The primary datasets used for this study are available at https://biolincc.nhlbi.nih.gov/studies/framoffspring/. Secondary data generated by image analysis is available from the corresponding author on reasonable request.
