# Supplementary material for: Cardiac microstructural alterations measured by echocardiography identify sex-specific risk for heart failure
Source: Heart. 2022 Jun 9;108(22):1800–6. doi: 10.1136/heartjnl-2022-320876 (PMC9626911; doi:10.1136/heartjnl-2022-320876)
Supplement: Supplementary data [file heartjnl-2022-320876supp001.pdf]

Cardiac Microstructural Alterations Measured by Echocardiography Identify Sex-Specific Risk for Heart Failure

Supplemental File

Correspondence: Alan C. Kwan, MD, Department of Cardiology, Smidt Heart Institute, Cedars-Sinai Medical Center, Los Angeles, CA; phone (310) 423-2726; email alan.kwan@cshs.org, and **Susan Cheng, MD, MPH, MMSc**, Department of Cardiology, Smidt Heart Institute, Cedars-Sinai Medical Center, Los Angeles, CA; phone (310) 423-2726; email biodatacore@cshs.org.

Kwan A, *et al. Heart* 2022; 108:1800–1806. doi: 10.1136/heartjnl-2022-320876

Supplemental Figure 1. Study sampling strategy.

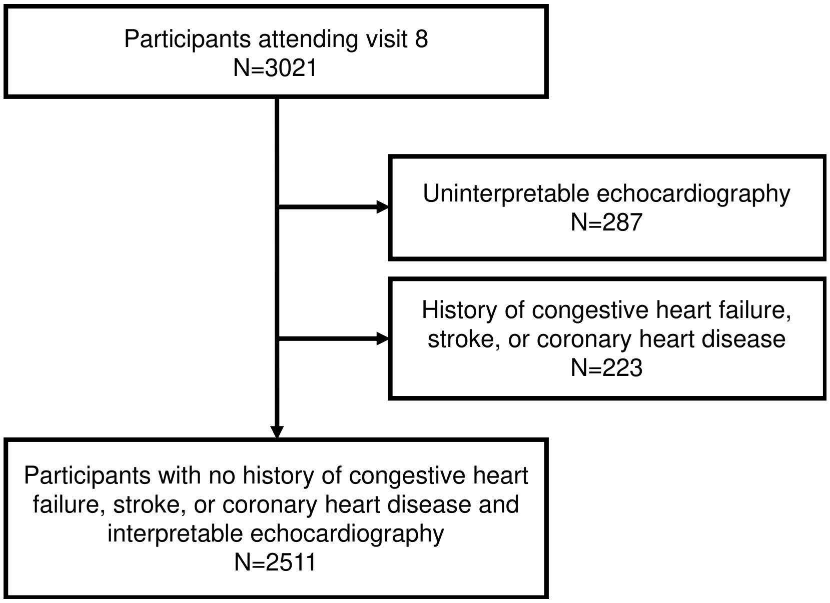

Kwan A, *et al. Heart* 2022; 108:1800–1806. doi: 10.1136/heartjnl-2022-320876

Supplemental Figure 2. Cumulative Incidence Curve for Survival Free from Heart Failure.

Panel A displays the cumulative incidence of heart failure free survival in participants stratified by high spectrum signal intensity coefficient (HS-SIC) where blue lines denote participants with HS-SIC in the top fourth and red lines denote participants with HS-SIC in lower three fourths.

Panel B displays the cumulative incidence of heart failure free survival in participants stratified by both HS-SIC and sex, where green lines denote women with HS-SIC in the lower three fourths, red lines denote men with HS-SIC in the lower three fourths, purple lines denote women with HS-SIC in the top fourth, and blue lines denote men with HS-SIC in the bop fourth.
*** P<0.001, **P<0.01, *P<0.05, NS = Not significant.

Kwan A, *et al. Heart* 2022; 108:1800–1806. doi: 10.1136/heartjnl-2022-320876

A.

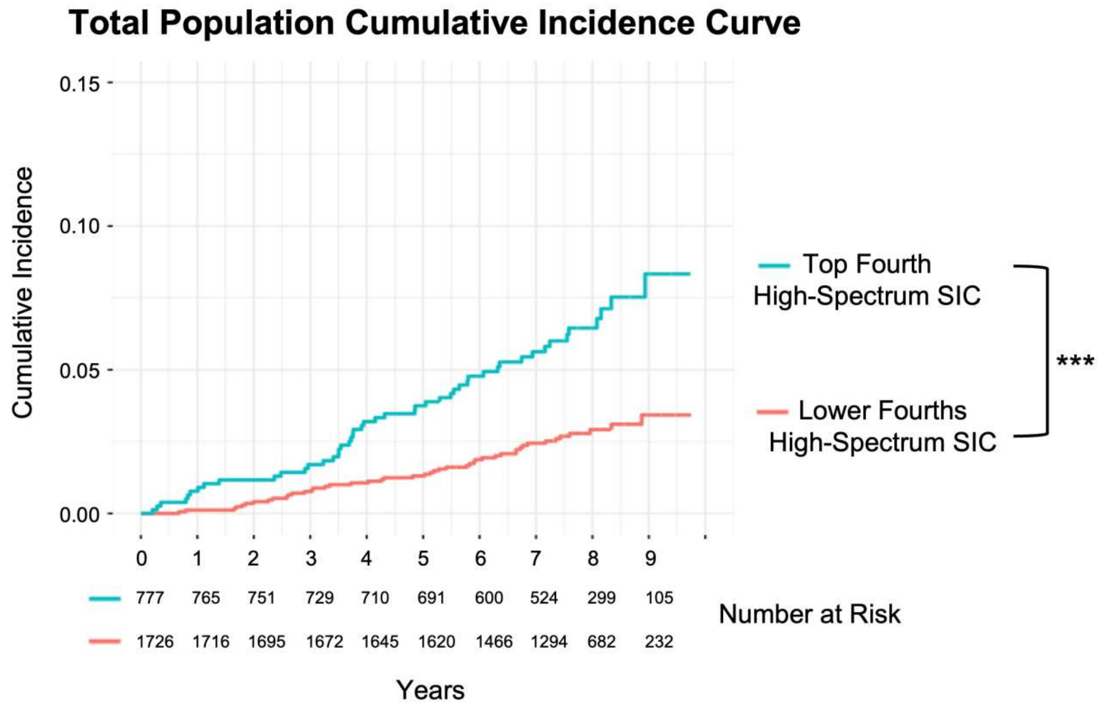
Total Population Cumulative Incidence Curve
Cumulative Incidence
0.15
0.10
0.05
0.00
0 1 2 3 4 5 6 7 8 9
Top Fourth High-Spectrum SIC
Lower Fourths High-Spectrum SIC
***
777 765 751 729 710 691 600 524 299 105
1726 1716 1695 1672 1645 1620 1466 1294 682 232
Number at Risk
Years

B.

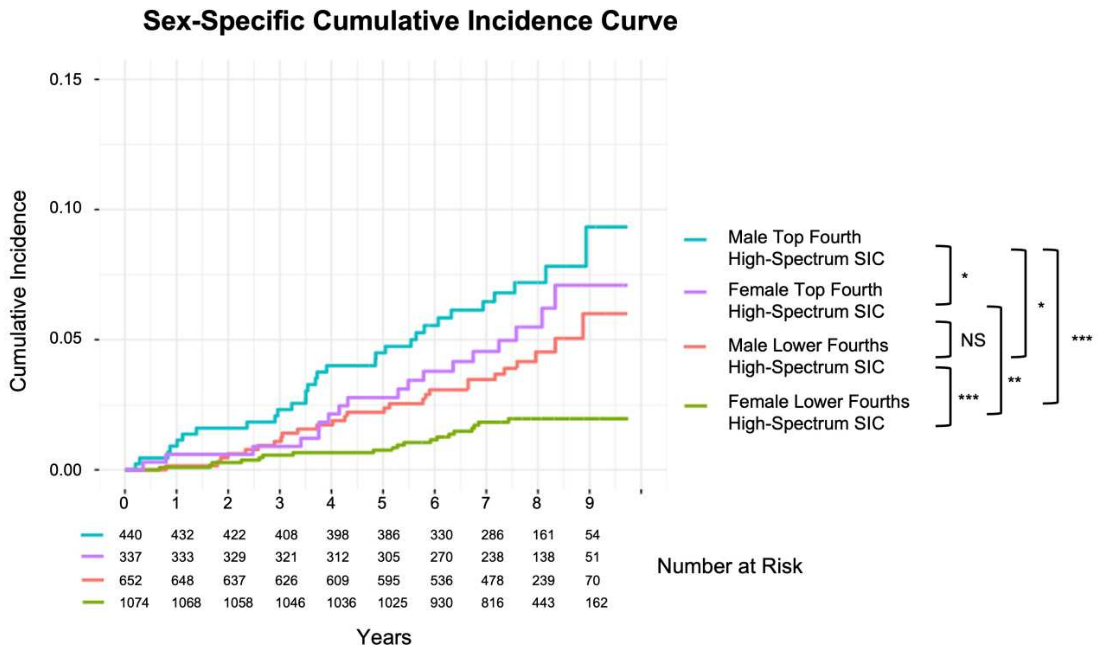
Sex-Specific Cumulative Incidence Curve
Cumulative Incidence
0.15
0.10
0.05
0.00
0 1 2 3 4 5 6 7 8 9
Male Top Fourth High-Spectrum SIC
Female Top Fourth High-Spectrum SIC
Male Lower Fourths High-Spectrum SIC
Female Lower Fourths High-Spectrum SIC
*
NS
***
*
**
***
440 432 422 408 398 386 330 286 161 54
337 333 329 321 312 305 270 238 138 51
652 648 637 626 609 595 536 478 239 70
1074 1068 1058 1046 1036 1025 930 816 443 162
Number at Risk
Years
